# Curcuminoid supplementation in canine diabetic mellitus and its complications using proteomic analysis

**DOI:** 10.3389/fvets.2022.1057972

**Published:** 2022-12-23

**Authors:** Namphung Suemanotham, Pongsakorn Photcharatinnakorn, Boonrat Chantong, Shutipen Buranasinsup, Sataporn Phochantachinda, Walasinee Sakcamduang, Onrapak Reamtong, Tipparat Thiangtrongjit, Duangthip Chatchaisak

**Affiliations:** ^1^Department of Clinical Sciences and Public Health, Faculty of Veterinary Science, Mahidol University, Nakhon Pathom, Thailand; ^2^Department of Pathology, Faculty of Veterinary Science, Chulalongkorn University, Bangkok, Thailand; ^3^Faculty of Veterinary Science, Prasu Arthorn Animal Hospital, Mahidol University, Nakhon Pathom, Thailand; ^4^Department of Pre-clinic and Applied Animal Science, Faculty of Veterinary Science, Mahidol University, Nakhon Pathom, Thailand; ^5^Department of Molecular Tropical Medicine and Genetics, Faculty of Tropical Medicine, Mahidol University, Bangkok, Thailand

**Keywords:** curcuminoids, diabetes mellitus, inflammation, oxidative stress, proteomic

## Abstract

**Introduction:**

Inflammation and oxidative stress contribute to diabetes pathogenesis and consequences. Therapeutic approaches for canine diabetes remain a challenge. Curcumin has anti-inflammatory and anti-oxidative effects and is beneficial for humans with diabetes mellitus (DM); however, data on its impact on canine diabetes is limited. This study aimed to evaluate the potential for causing adverse effects, anti-inflammatory effects, anti-oxidative effects and proteomic patterns of curcuminoid supplementation on canine DM.

**Methods:**

Altogether, 18 dogs were divided into two groups: DM (*n* = 6) and healthy (*n* = 12). Curcuminoid 250 mg was given to the DM group orally daily for 180 days. Blood and urine sample collection for hematological parameters, blood biochemistry, urinalysis, oxidative stress parameters, inflammatory markers and proteomics were performed every 6 weeks.

**Results and discussion:**

Curcuminoid supplementation with standard therapy significantly decreased oxidative stress with the increased glutathione/oxidized glutathione ratio, but cytokine levels were unaffected. According to the proteomic analysis, curcuminoid altered the expression of alpha-2-HS-glycoprotein, transthyretin, apolipoprotein A-I and apolipoprotein A-IV, suggesting that curcuminoid improves insulin sensitivity and reduces cardiovascular complications. No negative impact on clinical symptoms, kidneys or liver markers was identified. This study proposed that curcuminoids might be used as a targeted antioxidant strategy as an adjunctive treatment to minimize diabetes complications in dogs.

## 1. Introduction

Diabetes mellitus (DM) is a disorder that disrupts the body from shifting glucose into the cells resulting in hyperglycaemia (1). Insulin dependence or DM that resembles type I diabetes in humans is commonly observed in dogs (2). Canine DM diagnosis is based on fasting hyperglycaemia and glucosuria with clinical presentation of polyuria, polydipsia, polyphagia and weight loss (2). Its treatment goal is blood glucose control, which can be accomplished through insulin therapy, dietary modification and control of concurrent disorders (3). The major complications of DM include diabetic nephropathy, diabetic neuropathy, diabetic retinopathy, diabetic cardiomyopathy and atherosclerosis induced by chronic hyperglycaemia *via* several pathways (4). The proposed unifying mechanism that mediates the tissue-damaging effects of hyperglycaemia is superoxide overproduction (5).

Effective monitoring is required for DM treatment to reduce the risk of progression and complication. Hence, the identification of novel biomarkers is being researched (6, 7). Proteomics has been recognized as an important tool for establishing a diagnosis of disease etiology and monitoring therapy outcomes (8, 9). Proteomic patterns were applied to detect diabetes and complications, as well as to evaluate treatment effectiveness in humans (10–12). There is limited information on proteomic data in DM dogs (13–15). In a proteomic analysis of serum samples from DM dogs, most up-regulated proteins are involved in oxidative state, defense and inflammation (13).

Medicinal plants are utilized in DM dogs as an adjunct medicine in combination with standard treatment to prevent the development of long-term diabetes complications and improve overall wellbeing. Curcumin, the most phytochemically active curcuminoid extracted from *Curcuma longa*, has gained attention in human and laboratory animals. Curcumin is known to have antioxidant, anti-inflammatory and anticancer properties (16–18). In humans and experimental animals with DM, curcumin has an antioxidant potential of enhanced reduced glutathione (GSH) and reduced malondialdehyde (MDA) levels (19, 20). The anti-inflammatory effects of curcumin in DM were reported *via* decreased interleukin-1β (IL-1β), interleukin-6 (IL-6), interleukin-8 (IL-8) and tumor necrosis factor-α levels and also diminished monocyte chemoattractant protein-1 and C-reactive protein levels (19–21).

There has been no published evidence of the impact, adverse effects or proteomic profiles of curcuminoids, particularly curcumin, in client-owned DM dogs. Accordingly, the aims of the present study were (1) to evaluate the effects of curcuminoid supplementation on canine DM-associated oxidative stress and inflammation, (2) to determine the potential for causing adverse effects of curcuminoid supplementation in canine diabetes and (3) to determine whether curcuminoid has an impact on proteins implicated in DM-associated complications by a proteomic analysis.

## 2. Materials and methods

### 2.1. Animals

Altogether, 18 client-owned dogs from Prasuarthorn Hospital, Faculty of Veterinary Science, Mahidol University, Thailand were recruited into the study and then divided into two groups: DM (*n* = 6) and clinically healthy (*n* = 12) dogs. For the DM group, blood collection on day 0 is taken as sample prior to supplementation of curcuminoid. The comparison of this sample is performed to determine the blood biochemical parameters of healthy dog vs. DM dog that has not been given supplementation (“untreated”).

DM was diagnosed in dogs with fasting hyperglycemia, a high blood fructosamine level and glucosuria. The inclusion criteria for DM were dogs of any breed, age or sex, which had a stable blood glucose level for at least 3 months. The diabetic dogs with complications that affected their blood glucose level, including diabetic ketoacidosis, acromegaly, exocrine pancreatic insufficiency or neoplasia, were excluded to prevent confounding factors that would affect parameters and data analysis. Dogs over the age of 7 years of any breed or sex with normal vital signs and blood test results were used as the clinically healthy group for a cross-sectional well-being baseline evaluation.

The DM dogs received routine treatment combined with oral turmeric extract capsule (Antiox, Government Pharmaceutical Organization, Thailand, lot: CAO 63036) supplementation once daily either with meal or immediately after meal for 180 days. Each capsule contained 250 mg of curcuminoids.

The owners were informed and signed in the consent form. The study was approved by the Committee on the Care and Use of Laboratory Animals in the Faculty of Veterinary Science, Mahidol University, Thailand (approval number: MUVS-2020-04-10).

### 2.2. Samples

In the DM group, clinical parameters of hematology, biochemistry, blood glucose, serum fructosamine and urinalysis were evaluated every 6 weeks from 0 to day 180. Oxidative stress, inflammation and proteomic markers were measured in the DM group on days 0 and 180. Blood samples were collected from either the cephalic or the saphenous vein in a volume of at least 5–9 ml. One drop of blood was immediately tested for glucose concentration using a glucometer (AlphaTRAK, Zoetis, Parsippany, NJ, USA). Ethylenediaminetetraacetic acid (EDTA) blood samples were kept at 4°C, and hematological indices were analyzed within 4 h using an animal blood counter (Mindray, Shenzhen, China). Plain tubes for the measurement of fructosamine concentration were processed within 24 h using an automatic analyser (DIRUI, Jilin, China).

Heparinized samples were centrifuged (Hettich Lab Technology, Tuttlingen, Germany) at 3,500 rpm for 5 min within an hour of blood collection to separate plasma. Aliquoted plasma samples were used to measure clinical biochemical parameters (alanine aminotransferase, alkaline phosphatase, blood urea nitrogen and creatinine levels) using an automatic analyser (Beckman Coulter, Brea, CA, USA). Plasma symmetrical dimethylarginine (SDMA) was quantified using a catalyst SDMA automatic analyser (IDEXX Laboratories, Westbrook, ME, USA). The remaining plasma samples were collected in sterile microcentrifuge tubes and stored at −80°C for the measurement of oxidative stress parameters [MDA, GSH and oxidized glutathione (GSSG)] and cytokines (IL-6 and IL-10) as well as for proteomic analysis.

Urine samples were collected by midstream voiding to reduce stress and were kept at 4°C. They were subjected to a urinalysis within 4 h. Urine specific gravity was tested by a refractometer (VETAnyMall, Nonthaburi, Thailand). The urine samples were then centrifuged (GEMMY INDUSTRIAL CORP., Taipei, Taiwan) at 1,500 rpm for 5 min. Supernatants were tested for physical and chemical parameters using the dipstick test (Mission, ACON Laboratories, San Diego, CA, USA). Urine sediments were evaluated under a light microscope (ZEISS, Jena, Germany).

### 2.3. Determination of oxidative stress and cytokines

MDA level was determined using a thiobarbituric acid approach, which quantified MDA reactive products, measuring the absorbance at 535 nm (22). The concentration of MDA was quantified using the standard curve, which was established by hydrolysis of 1,1,3,3-tetrametoxypropane and data were represented in μM.

The levels of reduced GSH and GSSG were measured using the 5,5'-dithiobis-(2-nitrobenzoic acid) /glutathione reductase recycling method (23, 24). The results were referred to as the GSH/GSSG ratio.

The concentrations of canine IL-6 ab193686 (Abcam, Cambridge, UK) and IL-10 ab193685 (Abcam, Cambridge, UK) in plasma were measured using enzyme-linked immunosorbent assay and were carried out precisely as recommended by the manufacturer.

### 2.4. Proteomics and data processing

Protein digestion and analysis of peptide patterns by nano liquid chromatography with tandem mass spectrometry (nano-LC-MS/MS) of serum were performed, as previously described (25). For peptide identification, each lane in SDS-PAGE was pooled by two sets of dog serum in the same volume. A trypsin-treated sample (resuspended in 0.1% formic acid) was subjected to a nano-LC system (Dionex Ulti-Mate 3000). Peptide separation was performed at a flow rate of 300 nL/min using a C18 column and the following elution gradient: 4% mobile phase B (80% acetonitrile in 0.1% formic acid) to 50% mobile phase A (0.1% formic acid in water) for 30 min. The eluent was infused into a micrOTOF-Q mass spectrometer (Bruker Daltonics Billerica, MA, USA). The mass spectra obtained by mass spectrometry (MS) and tandem mass spectrometry (MS/MS) covered the *m/z* mass ranges from 400 to 2000 and from 50 to 1,500, respectively.

The LC-MS/MS data files were converted into a mascot generic file (.mgf) format with Data Analysis 3.4 version software. Mascot daemon version 2.3.02 (Matrix Science) was used to merge the.mgf files and to identify the proteins from the UniProt database. *Canis lupus familiaris* was set for the taxonomy filter. A total of 151,819 sequences were used as entries. The Mascot search allowed up to one missed cleavage and a peptide tolerance of 0.8 Da. The tandem MS tolerance was set to 0.6 Da. Variable modifications included methionine oxidation and cysteine carbamidomethylation. Selected protein hits had a *P* ≤ 0.05. The identity of the proteins reported in this research had a 95% confidence level. The protein expression was quantified by peptide count analysis using the exponentially modified protein abundance index (emPAI) provided by Mascot. Differentially expressed proteins in at least two of the biological replicates were reported as protein alterations in each group. In comparative proteomics, a change in protein concentrations of either higher or lower than 1.5-fold is considered significant, and proteins are considered differentially expressed between groups, i.e., up- or down-regulated, respectively (26, 27). Processed protein-level data were analyzed using a range of software tools (PANTHER, STRING and STITCH) (www.string-db.org, http://pantherdb.org and http://stitch.embl.de, respectively; accessed on 30 July 2022).

### 2.5. Statistical analysis

Clinically healthy dogs were compared with DM dogs prior to curcuminoid supplementation (day 0) by F-test for analysis of variance (ANOVA). The clinical parameters of DM including blood glucose, blood fructosamine, complete blood count and biochemistry were compared between day 0 and days 45, 90, 135 and 180 using repeated measures ANOVA. Oxidative stress and inflammatory parameters were compared between days 0 and 180 using F-test for ANOVA. Statistical calculations were performed using the IBM SPSS version 19 and Prism 9.00 GraphPad. The difference between the treatment and control groups were considered significant when *P* < 0.05.

## 3. Results

### 3.1. DM dogs after curcuminoid supplementation

**Figure 1:**
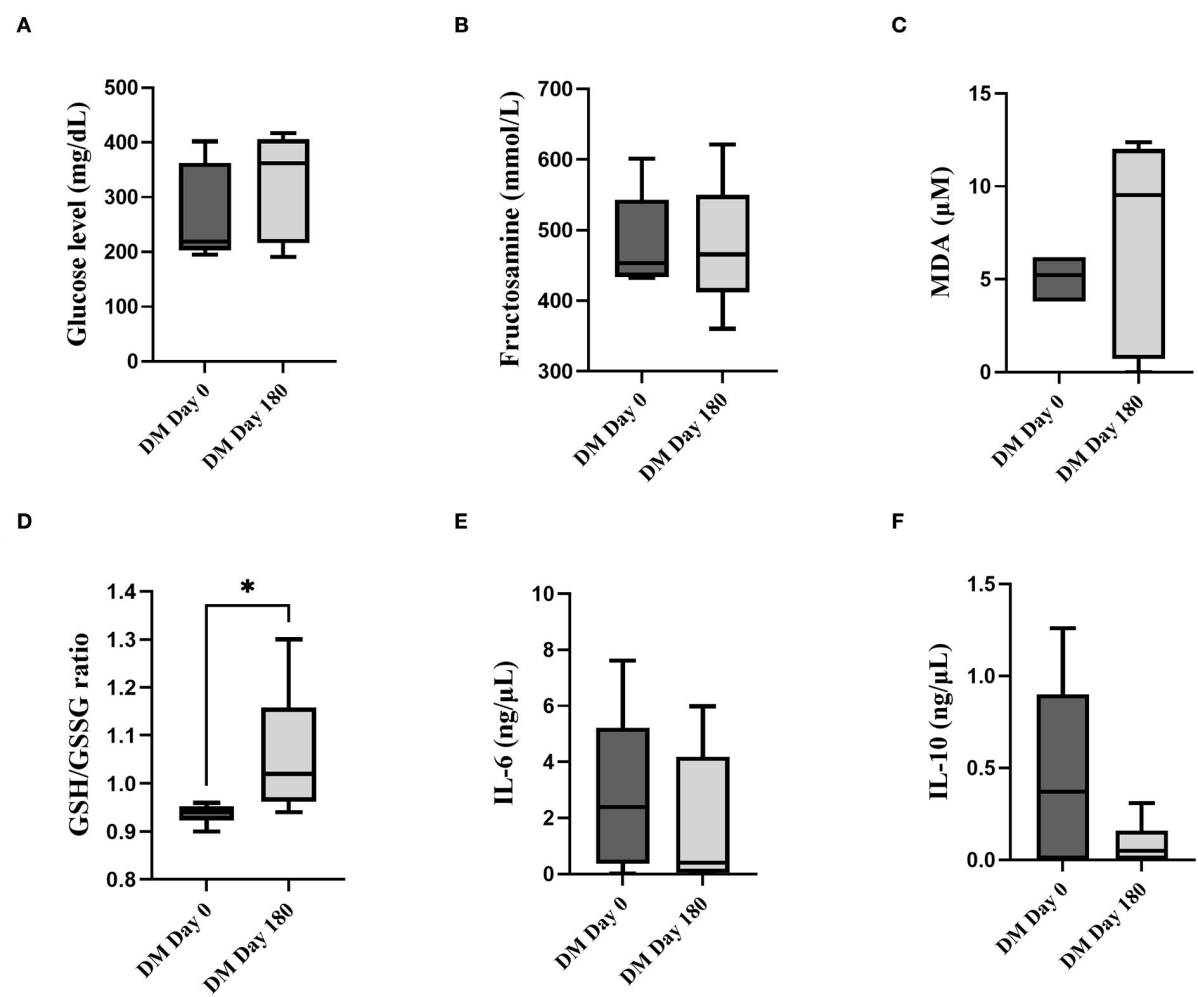
Comparison between the DM day 0 and 180 groups. **(A)** Fasting blood glucose (mg/dL). **(B)** Fructosamine (mmol/L). **(C)** MDA (μM). **(D)** GSH/GSSG. **(E)** IL-6 (ng/μL). **(F)** IL-10 (ng/μL) (*statistically significant at *P* < 0.05).

The parameters of clinically healthy and DM groups at day 0 were illustrated in Supplementary material A. Briefly, blood glucose and fructosamine levels were significantly higher in the DM day 0 group compare to the clinically healthy dogs (*P* < 0.05). The MDA level and GSH/GSSG ratio were significantly lower in the DM day 0 group compared to the clinically healthy dogs (*P* < 0.05). The fasting blood glucose (Figure 1A), fructosamine (Figure 1B), MDA (Figure 1C), IL-6 (Figure 1E) and IL-10 (Figure 1F) levels were not significantly different (*P* > 0.05) between DM day 0 and DM 180 dogs. When compared with the DM day 0 group, the DM day 180 group showed significantly increased GSH/GSSG ratio (Figure 1D) (*P* < 0.05).

All clinical blood parameters of the DM dogs did not significantly change (*P* > 0.05) at days 0, 45, 90, 135 and 180 after curcuminoid supplementation (Supplementary material A). The urinalysis results from day 0 to day 180 illustrated a negative ketone body (Supplementary material B). Additionally, there was no adverse effect of curcuminoids reported by the owners throughout the study period.

### 3.2. Proteomic profile

**Figure 2:**
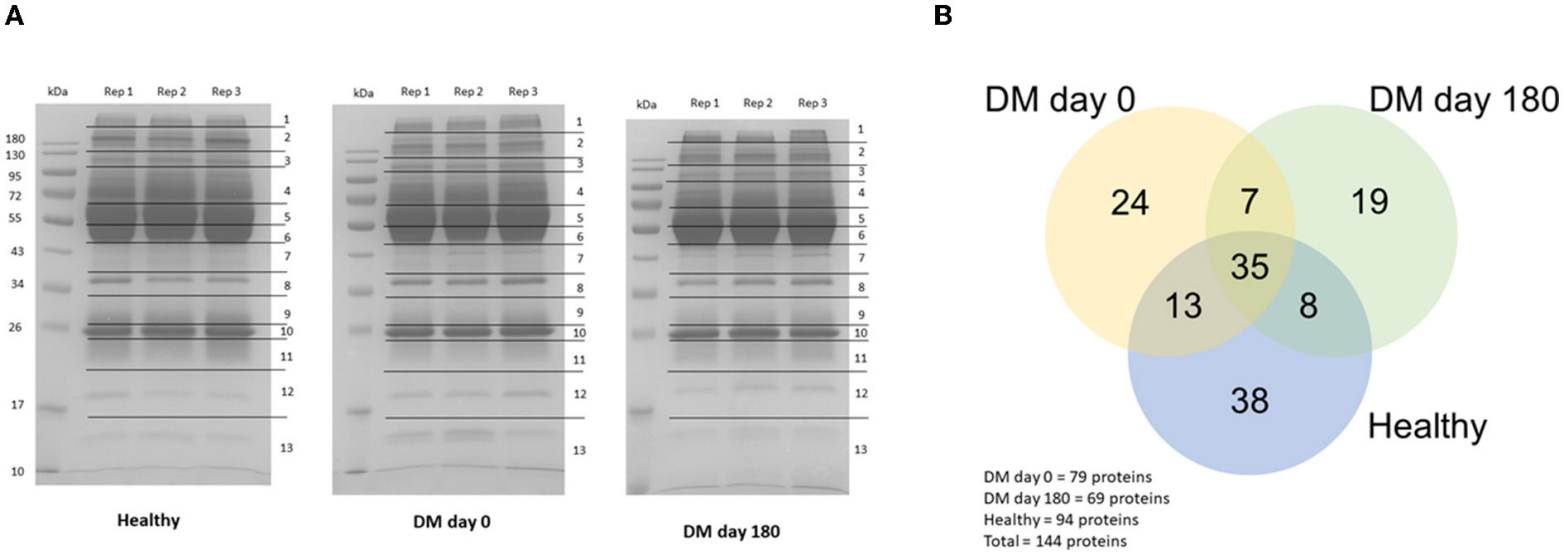
Protein detection. **(A)** 1D-Gel electrophoresis with Coomassie blue R-250 staining. **(B)** Venn diagram (*Canis lupus familiaris*).

The proteins were separated by 1D-gel electrophoresis. Nano-LC-MS/MS was used to identify the digested peptides. One hundred and forty-four proteins from the plasma samples were identified using the UniProt database. The protein bands of the DM day 0 group in rows 4, 12 and 13 were generally distinct from those of the healthy and DM day 180 groups (Figure 2A). All of the proteins from the three groups (healthy, DM day 0 and DM day 180) were visualized by using a Venn diagram analysis. Thirty-five proteins (24.30%) were found in all three groups when 144 canine proteins were compared. Forty-eight proteins (33.33%) were identified intersecting between the DM day 0 and healthy groups and 42 proteins (29.17%) between the DM day 0 and 180 groups (Figure 2B).

### 3.3. Expression of proteins

Comparative proteomic analysis was conducted to determine any alteration. Table 1 demonstrates that 28 proteins (8 up-regulated and 20 down-regulated) showed a differential expression in the plasma of the DM day 0 group, as compared to the healthy group. Table 2 shows that 25 proteins (13 up-regulated and 12 down-regulated) were differentially expressed in the plasma of the DM day 0 group, as compared to the DM day 180 group. The proteins with a differential expression in each of the two biological replicates with fold changes > 1.5 (up-regulated) and < 0.66 (down-regulated) were taken into consideration.

**Table 1:** Comparison of the protein fold change between the DM day 0 and healthy groups.

Accession number^*^	**Protein**	**Protein** **mass**	**Protein** **pI**	**Folds** **change**	**Description**
A0A5F4D5S2	C4a anaphylatoxin	190,522	6.33	4.33	Up-regulated
A0A5F4CMC0	Transferrin	78,055	6.46	3.88	Up-regulated
E2QUV3	Alpha-2-HS-glycoprotein	39,223	5.12	2.25	Up-regulated
A0A5F4BVF9	Apolipoprotein H	37,256	8.54	2.11	Up-regulated
A0A5F4BS70	Peptidase S1 domain-containing protein	34,360	5.78	-^**^	Up-regulated
A0A5F4D9G5	Joining chain of multimeric IgA and IgM	17,628	4.61	-^**^	Up-regulated
Q7M321	Plasmin (Fragments)	13,478	6.38	-^**^	Up-regulated
A0A5F4BRF0	Transferrin	74,413	8.63	-^**^	Up-regulated
E2RE31	Immunoglobulin heavy constant mu	52,952	5.53	7.33	Down-regulated
A0A5F4BZ99	Uncharacterised protein	164,176	6.37	6.5	Down-regulated
A0A5F4C2N3	Kininogen 1	51,067	5.95	4.67	Down-regulated
F1PGM9	Complement component 4 binding protein alpha	68,505	7.77	3.2	Down-regulated
F1PG16	CD5 molecule like	37,673	5.45	2.28	Down-regulated
A0A5F4D5F7	Serpin family A member 1	44,944	6.06	2.14	Down-regulated
A0A5F4C9M5	Piccolo presynaptic cytomatrix protein	520,825	5.92	-^***^	Down-regulated
A0A5F4BYG2	Ig-like domain-containing protein	12,529	8.56	-^***^	Down-regulated
A0A5F4CHV4	Alpha fetoprotein	69921	5.59	-^***^	Down-regulated
A0A1K0GGH0	Globin A2	16,117	7.83	-^***^	Down-regulated
A0A5F4CSY2	Apolipoprotein E	36,582	5.41	-^***^	Down-regulated
A0A5S6CZL3	Apolipoprotein A-IV	45,647	6.16	-^***^	Down-regulated
J9NUI6	Alpha-1-microglobulin	38,832	5.96	-^***^	Down-regulated
E2RS75	C3/C5 convertase	91,770	8	-^***^	Down-regulated
G1K2D9	Haptoglobin	38,358	5.83	-^***^	Down-regulated
F1PTY1	Cytokeratin-1	112,360	5.35	-^***^	Down-regulated
A0A5F4CF56	ATP binding cassette subfamily B member 7	78,301	9.58	-^***^	Down-regulated
A0A5F4DAK7	Uncharacterised protein	25,312	6.31	-^***^	Down-regulated
Q6EIY9	Keratin, type II cytoskeletal 1	63,751	7.66	-^***^	Down-regulated
E2R917	Keratin 75	60,212	6.95	-^***^	Down-regulated

* Accession number from UniProt database against Canis lupus familiaris,

** only found in DM day 0 group,

*** only found in healthy group.

**Table 2:** Comparison of the protein fold change between the DM day 180 and DM day 0 groups.

Accession number^*^	**Protein**	**Protein** **mass**	**Protein** **pI**	**Folds** **change**	**Description**
F1PDJ5	Apolipoprotein A-I	30,163	5.28	5.93	Up-regulated
E2RE31	Immunoglobulin heavy constant mu	52,952	5.53	3.33	Up-regulated
F1PGM9	Complement component 4 binding protein alpha	68,505	7.77	2.6	Up-regulated
A0A5F4BYJ5	Uncharacterised protein	50,268	8.17	2.41	Up-regulated
P01784	Ig heavy chain V region GOM	12,422	5.24	2.25	Up-regulated
F1PG16	CD5 molecule like	37,673	5.45	2.22	Up-regulated
A0A5F4CHV4	Alpha fetoprotein	69,921	5.59	-^**^	Up-regulated
F6V1W9	Transferrin	77,412	8.52	-^**^	Up-regulated
A0A5S7EUL7	Apolipoprotein E	38,512	5.35	-^**^	Up-regulated
A0A5F4C586	Transthyretin	17,966	6.95	-^**^	Up-regulated
F1PIB4	LDL receptor related protein 2	520,245	4.91	-^**^	Up-regulated
A0A5F4D9G5	Joining chain of multimeric IgA and IgM	17,628	4.61	7.37	Down-regulated
E2QUV3	Alpha-2-HS-glycoprotein	39,223	5.12	4.75	Down-regulated
A0A5F4CMC0	Transferrin	78,055	6.46	4.57	Down-regulated
A0A5F4D5S2	C4a anaphylatoxin	190,522	6.33	3.5	Down-regulated
A0A5F4DFV1	Apolipoprotein C-III	10,982	5.21	2.35	Down-regulated
A0A5B8JID5	Hemoglobin subunit alpha1	15,369	7.86	2.23	Down-regulated
E2RRM2	Prothrombin	70,259	5.71	2	Down-regulated
F1PGM1	C3-beta-c	176,028	6.74	1.5	Down-regulated
A0A5F4DFF1	Uncharacterised protein	161,041	6.44	-^***^	Down-regulated
A0A5F4CBH6	Ig-like domain-containing protein	11,245	8.03	-^***^	Down-regulated
A0A5F4CED7	Plasma retinol-binding protein	36,267	8.94	-^***^	Down-regulated
A0A5F4BRF0	Transferrin	74,413	8.63	-^***^	Down-regulated
Q7M321	Plasmin (Fragments)	13,478	6.38	-^***^	Down-regulated

* Accession number from UniProt database against Canis lupus familiaris,

** only found in the DM day 180 group,

*** only found in the DM day 0 group.

### 3.4. Gene ontology

**Figure 3:**
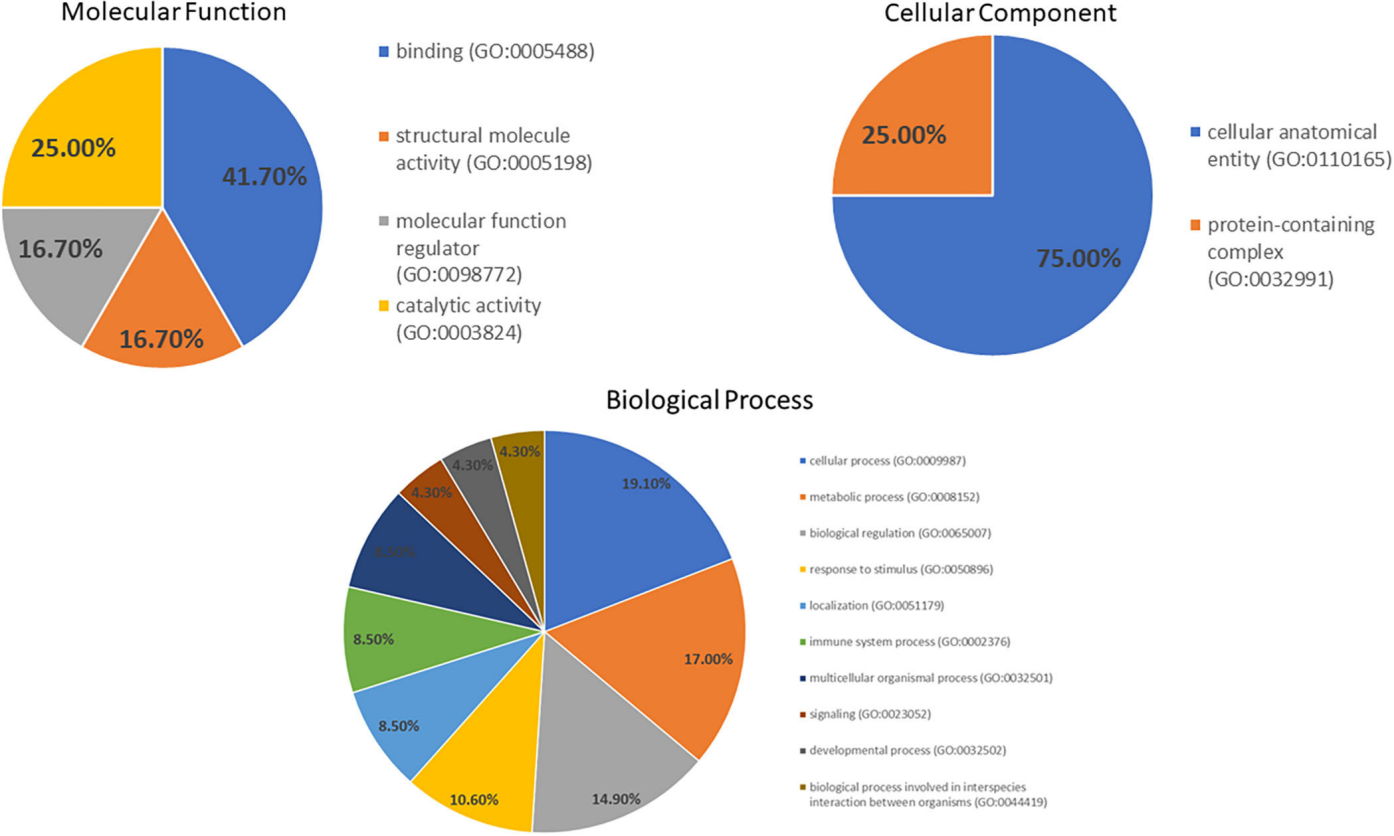
Gene ontology of the molecular function, cellular component and biological process categories for proteins' differential expression in the DM day 0 group, as compared to other groups.

A GO analysis of molecular function by PANTHER (Figure 3) revealed the proteins are engaged in binding (41.7%), catalytic (25.0%), structural molecular (16.7%) and molecular function regulator (16.7%) activities. The proteins in the biological processes were engaged in cellular processes (19.1%), metabolic processes (17.0%), biological regulation (14.9%), response to stimulus (10.6%) and other processes. Moreover, the primary cellular component was a cellular anatomical entity (75.0%).

### 3.5. Pathway enrichment analyses of proteins

**Figure 4:**
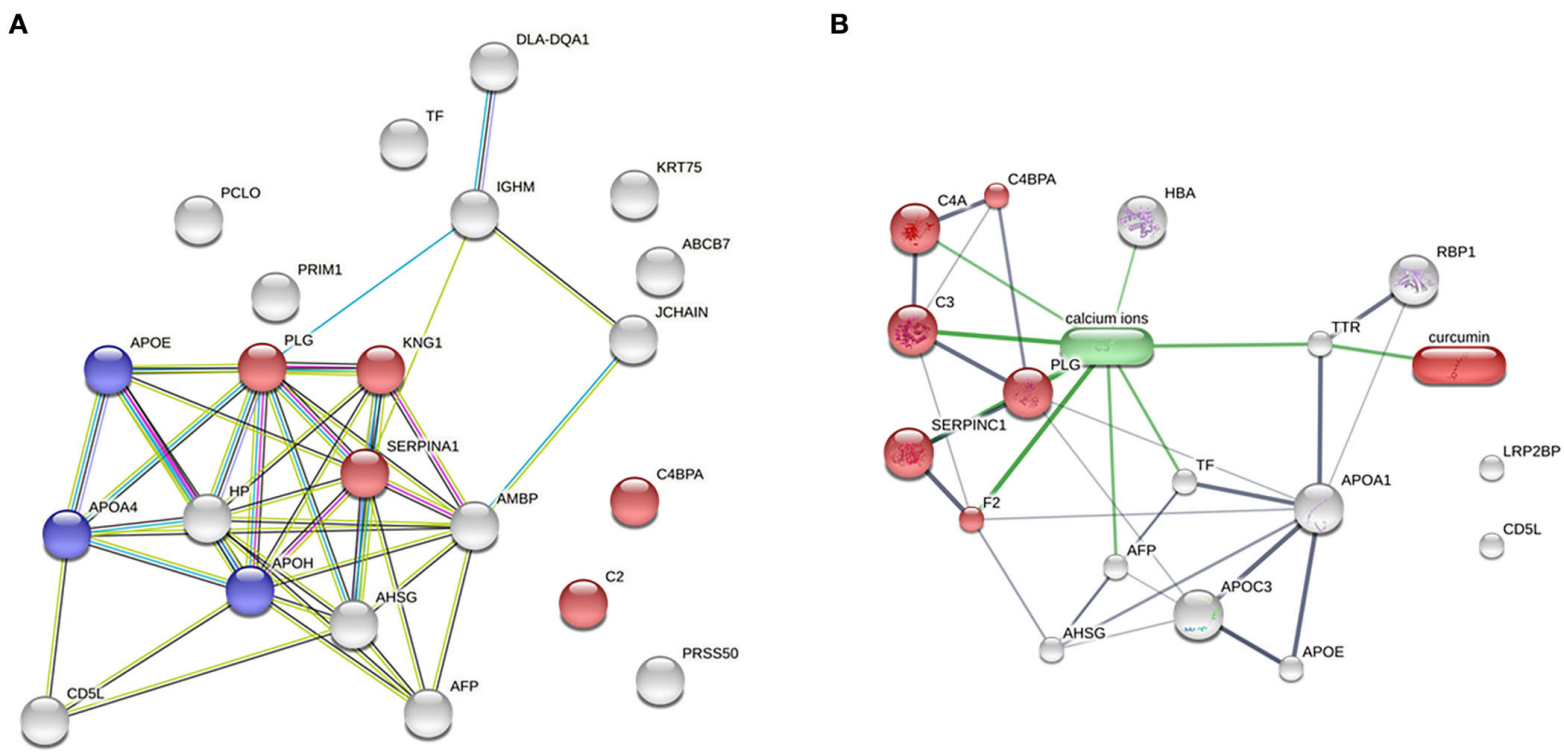
Network analysis. **(A)** Analysis of protein–protein interaction between the DM day 0 groups, as compared with the healthy group (total proteins = 22; red = complement and coagulation cascades; blue = cholesterol metabolism). **(B)** Relationship of curcumin–protein interaction between the DM day 0 group, as compared with the DM day 180 group (red = complement and coagulation cascades).

To demonstrate the evidence of protein–protein interaction by the STRING programme, 22 protein alterations of the DM day 0 group compared with the healthy group were expanded. The coagulation cascade and complement to cholesterol metabolism are the two main proteins involved (Figure 4A). In a network of drug–protein interaction by the STITCH programme, the edge confidence scores were used to determine how strong these pathway linkages were at the functional level. Thick lines are used to depict interactions with high-edge confidence scores (> 0.700). Transthyretin (TTR) was found to have a relationship with curcumin and a strong relationship with apolipoproteins (Figure 4B).

## 4. Discussion

DM causes hyperglycaemia, oxidative damage and inflammation. Curcumin has been demonstrated to decrease blood glucose levels and improve insulin resistance in patients with DM, as well as have anti-inflammatory and anti-oxidative properties, although clinical research in canine diabetes is limited (16, 28). The current research explored the influence of curcuminoid supplementation on canine DM-associated oxidative stress and inflammation, its adverse effects in canine diabetes and its impact on DM-correlated complication proteins by proteomic analysis.

In the current study, diabetic dogs were given curcuminoids at a dose of 250 mg/day, which was estimated using the approach described by Nair and Jacob (29), with conversion factors based on the dosage supplied, as reported in a clinical study (30). After 180 days of curcuminoid administration, the blood glucose parameters, including serum glucose, serum fructosamine and urine glucose, of DM dogs before and after 180 days of curcuminoid administration were unchanged. Curcuminoids may cause vomiting, diarrhea and allergies (31). The owners never reported these clinical signs. Following 180 days of oral curcuminoid administration, clinical biochemical indicators showed that the liver and kidney functions were unaltered. These results suggest that curcuminoids were safe for renal and hepatic functions when administered at a particular dose during the study's trial period used in this study. Our results also supported the findings of a prior investigation in canines with osteoarthritis, which revealed no negative effects following supplementation with turmeric extract (32). In rabbits, conversely, the administration of *Curcuma longa* (13 g/kg) for 90 days resulted in liver and kidney degeneration and necrosis, along with elevated clinical biochemical parameters (33).

Hyperglycaemia triggers factors that might enhance systemic oxidative stress and inflammation, with endothelial dysfunction as the underlying cause of micro- and macrovascular complications (34, 35). In the current research, increased MDA levels and lowered GSH/GSSG ratio were detected in DM dogs, indicating oxidative damage and limited antioxidant activity. Comparing healthy and DM dogs, there was a significant increase in inflammatory (IL-6) cytokine, but not in anti-inflammatory (IL-10) cytokines, which is consistent with recent reports demonstrating that DM is a chronic inflammatory disorder (36). The current study provided the first evidence that curcuminoids improved the GSH/GSSG ratio in DM dogs. These findings supported clinical evidence showing that curcumin lowered oxidative stress markers in humans with diabetes (16, 28).

The potential anti-oxidative properties of curcuminoids in canine DM were subsequently investigated to determine which possible target proteins curcuminoids may affect. In the serum proteomic analysis, 42 proteins were found to be altered in the DM group compared with the control and treatment groups. According to the PANTHER classification system, binding was the most represented molecular function [alpha-2-HS-glycoprotein (A2HS), retinol-binding protein-4 (RBP4), apolipoprotein A-IV (ApoA-4) and Ig heavy chain]. Similarly, the cellular process was the biological process most represented [A2HS, apolipoprotein E (ApoE), ApoA-4, transthyretin (TTR), haptoglobin, Ig heavy chain and keratin]. In a previous plasma proteomics study, C4b-binding protein beta chain, maltase-glucoamylase and haptoglobin heavy chain are up-regulated in DM dogs, as compared to healthy dogs (13). In the enrichment analysis, differentially expressed plasma proteins are involved in cholesterol metabolism, complement and coagulation cascades. This demonstrated that DM was associated with peripheral organ inflammation and cholesterol metabolism partly influenced by apolipoproteins. Particularly, A2HS, TTR, ApoA-1 and ApoA-4 were the five major proteins modified by curcuminoids and involved in the pathogenesis of canine DM.

C4a anaphylatoxin is a 77-amino acid peptide generated from complement C4 through the activation of the complement system. C4a's functional profile is unknown; however, anaphylatoxins can cause inflammation as severe as type I hypersensitivity allergic responses (37). C4a activated ERK1/2 and enhanced endothelial permeability (38). Although no direct connection between C4a levels and DM pathophysiology has been established, high levels of C4a were found in patients with cardiovascular problems (39, 40). The present investigation revealed the upregulation of C4a in DM, and the curcuminoid treatment lowered the C4a level, suggesting that curcuminoids may reduce cardiovascular complications in DM by reducing the C4a level.

Transthyretin (TTR) is a plasma and cerebrospinal fluid transport protein that transports thyroxine and retinol and is recognized as a negative acute-phase protein (41). TTR was shown to be negatively associated with inflammation and oxidative stress (42). In human and rat studies, DM had lower TTR levels than the control group (43). In the current investigation, plasma TTR levels were not found in either the DM or healthy group. Interestingly, curcuminoids increased the TTR levels in the DM groups. The STITCH drug–protein interaction demonstrated that curcumin impacts TTR and contributes to the various proteins, including apolipoprotein A-I (ApoA-1), ApoA-4, ApoE and LDL receptor related protein-2 (LRP2). Curcuminoid's protective effect suggests that it prevents TTR misfolding and degradation (43–45).

Alpha-2-HS-glycoprotein (A2HS), or fetuin-A, is a cysteine protease inhibitor generated and secreted by the liver (46). A2HS is also a toll-like receptor-4 activator linked to insulin resistance and inflammation (47, 48). Additionally, A2HS induced insulin resistance by inhibiting insulin receptor tyrosine kinase (49, 50). Serum A2HS levels were shown to have a positive correlation with chronic hyperglycaemia, as well as insulin resistance, glucose tolerance, circulating lipid levels and obesity (47, 51). A2HS was up-regulated in canine DM in the present study, similar to the results of previous studies in humans with DM (46). Notably, DM dogs given curcuminoids had lowered A2HS levels, implying that curcuminoids minimizes the potential of insulin resistance by decreasing the A2HS levels.

ApolipoproteinA-1 (ApoA-1) is high density lipoprotein's primary component. Lipid-free and lipid-associated ApoA-1 increased insulin secretion and lowered the plasma glucose levels (52). The ApoA-1 levels were lower in patients with DM than in controls and were associated with diabetic retinopathy severity (53, 54). ApoA-1 decreased plaque inflammation and atherosclerosis in diabetic mice (55). ApolipoproteinA-4 (ApoA-4) is a glycoprotein found in chylomicrons, HDL and very low-density lipoprotein. In its lipoprotein-free form, it exhibits anti-atherogenic characteristics (56). A low plasma ApoA-4 level is associated with coronary artery disease in type 1 DM (57). Several studies have shown that ApoA-4 increased insulin secretion, sensitivity and glucose absorption (58–60). In the current study, the ApoA-1 levels in the healthy and DM groups were similar, whereas ApoA-4 was down-regulated in the DM group. ApoA-1 and ApoA-4 were elevated by curcuminoids. These data suggest curcuminoids may ameliorate diabetes and cardiovascular complications by enhancing ApoA-1 and ApoA-4 levels.

In this study, we used proteomics to identify proteins implicated in the etiology of canine DM that MDA and cytokine levels could not. We also presented the possibility of target proteins for curcuminoids intervention in diabetes, which can lead to categorizing most of the components, functionalising specific proteins, presenting the parts into relevant networks. This research has several limitations. Our findings must be verified and validated in larger cohorts owing to the study's small sample size. Another constraint is that the type of DM in dogs should be identified. The type of food, amount of feeding and insulin administration schedule were uncontrolled by dog owners during the study. These uncontrolled factors were commonly found in the clinical trial and might influence the experimental results.

**Figure 5:**
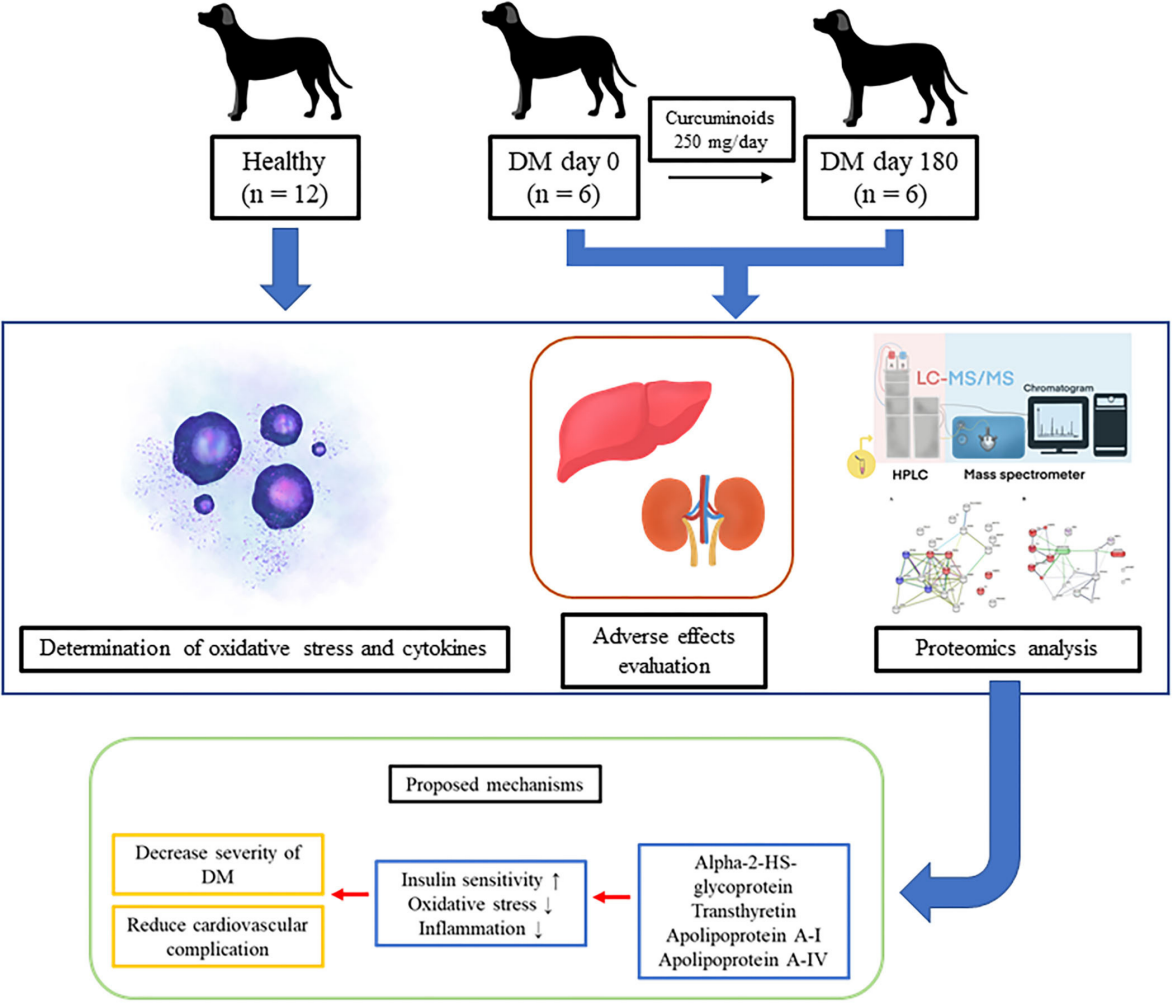
The improvement of the metabolic pathways influencing insulin sensitivity and cardiovascular events by curcuminoid supplementation in diabetic dogs.

This is the first evidence to show that curcuminoids can reduce oxidative stress and also have a relevance on the proteomic profile of DM dogs. Curcuminoid supplementation at a dose of 250 mg/day for 180 days enhanced the plasma GSH/GSSG ratio and improved the pathways influencing insulin sensitivities and cardiovascular events (Figure 5). No adverse effects on the clinical signs as well as kidney and liver parameters were observed. Therefore, we suggest that using curcuminoids might hold promise as a targeted antioxidant approach as an alternative adjunct therapy to reduce diabetic complications in dogs. However, further research must apply organ-specific proteomics to identify potential biomarkers and gain understanding into DM development processes. Using proteomics and natural supplements may facilitate the development of curcuminoids as a promising phytomedicine product for canine DM.

## Data availability statement

The datasets presented in this study can be found in online repositories. The names of the repository/repositories and accession number(s) can be found here: https://www.ebi.ac.uk/pride/, PXD037667.

## Ethics statement

The animal study was reviewed and approved by the Committee on the Care and Use of Laboratory Animals in the Faculty of Veterinary Science, Mahidol University, Thailand (approval number: MUVS-2020-04-10). Written informed consent was obtained from the owners for the participation of their animals in this study.

## Author contributions

NS, BC, and DC contributed to conception, supervised, data collection, and conducted data analysis. PP, SB, SP, and OR contributed to data curation. DC provided funding acquisition. NS, PP, SB, SP, and WS performed investigation. BC and DC designed methodology. OR and TT provided resources. PP, BC, WS, and DC conducted validation and visualization. NS, BC, SP, and DC prepared the original draft. NS, PP, BC, SB, SP, WS, OR, TT, and DC review and editing the manuscript. All authors have read and agree to the published version of the manuscript.
