# Supplementary material for: Curcuminoid supplementation in canine diabetic mellitus and its complications using proteomic analysis
Source: Front Vet Sci. 2022 Dec 23;9:1057972. doi: 10.3389/fvets.2022.1057972 (PMC9816143; doi:10.3389/fvets.2022.1057972)
Supplement: Supplementary file 1 [file Data_Sheet_1.docx]

**Supplementary material A: Clinical parameters**

*Diabetic and clinically healthy dogs before curcuminoids supplementation*

The clinically healthy dogs had significantly lower platelet number and higher creatinine level compared to DM dogs at day 0 (*P*<0.05). The other parameters at day 0 including WBC, RBC, BUN, SDMA, ALT, ALP and AST levels were not different between groups. Fasting blood glucose (Fig. 1A) and fructosamine (Fig. 1B) levels were significantly greater in the DM day 0 group at *P*<0.0001 and *P*<0.05, respectively. Compared to the clinically healthy, MDA (Fig. 1C) and GSH/GSSG ratio (Fig. 1D) were significantly lower in the DM day 0 group (*P*<0.05). The IL-6 level in diabetic dogs was significantly higher than in healthy dogs (Fig. 1E). IL-10 concentrations did not differ between groups (*P*>0.05) (Fig. 1F).


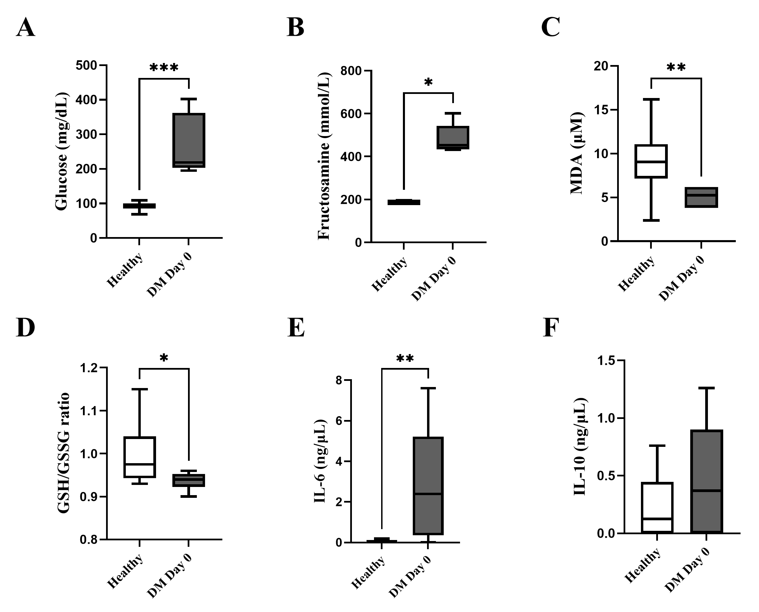


**Supplement Fig. 1** Comparison between healthy and DM day 0 groups (A). Fasting blood glucose (mg/dL) (B). Fructosamine (mmol/L) (C). MDA (µM) (D). GSH/GSSG, (E). IL-6 (ng/µL) (F). IL-10 (ng/µL) (****statistically significant at *P*<0.0001 and *statistically significant at *P*<0.05)

**Supplement Table 1** Clinical parameters of clinically healthy (n=12) and diabetic dogs (n=6) prior curcuminoids supplementation, *Statistically significant at *P*<0.05

| **Parameter** | **Reference range** | **Mean (percentile 25^th^-75^th^)** | | ***P*-value** |
| --- | --- | --- | --- | --- |
|  |  | **Healthy** | **DM day 0** |  |
| WBC x10^3^ (cell/mm^3^) | 6-17 | 10.49 (9.16-11.92) | 11.07 (9.91-12.36) | 0.60 |
| RBC x10^6^ (cell/mm^3^) | 5-9 | 6.61 (5.92-7.16) | 7.53 (7.00-7.59) | 0.17 |
| Platelet x10^3^ (cell/uL) | 200-500 | 157.50 (75.00-237.00) | 375.00 (297.75-402.00) | 0.00* |
| BUN (mg/dL) | 7-27 | 11.00 (8.00-12.00) | 12.00 (11.00-12.50) | 0.34 |
| Creatinine (mg/dL) | 0.50-1.80 | 1.05 (0.90-1.30) | 0.65 (0.58-0.70) | 0.00* |
| SDMA (ug/dL) | 0-14 | 13.00 (13.00-13.50) | 10.50 (7.75-13.50) | 0.36 |
| ALT (U/L) | 10-118 | 35.50 (32.00-59.00) | 52.50 (35.50-93.75) | 0.57 |
| ALP (U/L) | 20-150 | 78.50 (54.00-107.00) | 116.50 (96.25-210.25) | 0.33 |
| AST (U/L) | 14-45 | 30.00 (27.00-34.00) | 33.50 (31.75-37.50) | 0.94 |

**Supplement Table 2** Clinical parameters of kidney and liver in diabetic dogs supplemented with curcuminoids (n=6). Data were shown as mean ± SD. *Statistically significant at *P*<0.05

| **Parameter** | **Mean (percentile 25^th^-75^th^)** | | | | | ***P*-value** |
| --- | --- | --- | --- | --- | --- | --- |
|  | **DM day 0**  **(n=6)** | **DM day 45**  **(n=6)** | **DM day 90**  **(n=6)** | **DM day 135**  **(n=6)** | **DM day 180**  **(n=6)** |  |
| WBCx10^3^  (cell/mm^3^) | 11.07  (9.91-12.36) | 10.55  (8.10-14.62) | 13.25  (10.96-18.47) | 9.78  (7.63-12.21) | 10..96  (8.86-16.21) | 0.15 |
| RBC x10^6^(cell/mm^3^) | 7.53  (7.00-7.59) | 7.51  (6.73-7.81) | 7.12  (6.67-7.47) | 7.16  (6.86-7.36) | 7.27  (6.44-7.41) | 0.46 |
| Platelet x10^3^(cell/uL) | 375.00  (297.75-402.00) | 317.00 (284.00-322.50) | 299.00 (245.25-375.25) | 312.50 (248.00-401.75) | 380.00 (245.00-468.25) | 0.21 |
| BUN (mg/dL) | 12.00  (11.00-21.50) | 15.50  (11.75-21.25) | 15.00  (9.75-19.50) | 13.50  (11.75-18.25) | 15.50  (14.00-20.00) | 0.29 |
| Creatinine (mg/dL) | 0.65  (0.58-0.70) | 0.45  (0.38-0.65) | 0.55  (0.48-0.75) | 0.65  (0.48-0.85) | 0.50  (0.40-0.63) | 0.40 |
| SDMA (ug/dL) | 10.50  (7.75-13.50) | 9.50  (7.75-11.25) | 10.00  (8.75-13.00) | 9.00  (7.25-12.50) | 10.50  (7.75-13.00) | 0.79 |
| ALT (U/L) | 52.50  (35.50-93.75) | 56.00  (35.50-135.00) | 64.00  (33.00-122.25) | 54.00  (33.00-109.50) | 44.50  (30.00-82.25) | 0.31 |
| ALP (U/L) | 165.50 (96.25-210.25) | 129.00 (109.75-190.00) | 165.50 (103.75-227.75) | 153.00 (100.00-243.00) | 114.50 (100.50-223.25) | 0.95 |
| AST (U/L) | 33.50  (31.75-37.50) | 32.50  (25.50-45.50) | 32.00  (27.50-41.25) | 31.50  (28.00-35.25) | 30.00  (27.25-39.50) | 0.78 |
