# Supplementary material for: Curcuminoid supplementation in canine diabetic mellitus and its complications using proteomic analysis
Source: Front Vet Sci. 2022 Dec 23;9:1057972. doi: 10.3389/fvets.2022.1057972 (PMC9816143; doi:10.3389/fvets.2022.1057972)
Supplement: Supplementary file 2 [file Data_Sheet_2.docx]

|  | DM 1 | | | | | DM 2 | | | | | DM 3 | | | | |
| --- | --- | --- | --- | --- | --- | --- | --- | --- | --- | --- | --- | --- | --- | --- | --- |
|  | Day 0 | Day 45 | Day 90 | Day 135 | Day 180 | Day 0 | Day 45 | Day 90 | Day 135 | Day 180 | Day 0 | Day 45 | Day 90 | Day 135 | Day 180 |
| **Dipstick examination** | | | | | | | | | | | | | | | |
| USG | 1.045 | 1.032 | 1.038 | 1.034 | 1.048 | 1.016 | 1.018 | 1.017 | 1.016 | 1.030 | 1.030 | 1.035 | 1.035 | 1.038 | 1.033 |
| pH | 6 | 6 | 6 | 6 | 6 | 6 | 7 | 6 | 8.5 | 6 | 7 | 7.5 | 7.5 | 7 | 6 |
| Leukocyte | - | - | - | - | - | - | - | - | - | - | - | - | - | - | - |
| Nitrite | - | - | - | - | - | + | + | + | + | + | - | - | - | - | - |
| Protein | trace | trace | trace | - | trace | - | - | - | - | - | - | trace | trace | 1+ | trace |
| Glucose | 3+ | N | N | 3+ | 3+ | N | 3+ | N | N | 3+ | 3+ | 3+ | 3+ | 3+ | 3+ |
| Ketone | - | - | - | - | - | - | - | - | - | + | - | - | - | - | - |
| Urobilinogen | N | N | N | N | N | N | N | N | N | N | N | N | N | N | N |
| Bilirubin | - | - | - | - | - | - | - | - | - | - | - | - | - | - | - |
| Erythrocyte | - | - | - | - | - | - | - | - | - | - | - | - | - | - | - |
| **Microscopic Examination** | | | | | | | | | | | | | | | |
| WBC | 0-1 | NF | NF | NF | NF | NF | NF | 2-3 | NF | NF | NF | NF | NF | NF | NF |
| RBC | 0-1 | NF | NF | NF | NF | NF | NF | NF | NF | NF | NF | NF | 0-1 | NF | NF |
| Bacteria | few | 1+ | few | 1+ | few | 1+ | few | 2+ | 1+ | few | 1+ | 1+ | few | few | few |
| Amorphous | NF | NF | NF | NF | NF | NF | NF | NF | NF | NF | NF | NF | NF | NF | NF |
| Mucous | NF | NF | NF | NF | NF | NF | NF | NF | NF | NF | NF | NF | NF | NF | NF |
| epithelium | Sq 0-1 | Sq0-1 | NF | NF | NF | Sq0-1 | Sq0-1 | NF | NF | NF | Sq0-1 | Sq0-1 | Sq0-1 | NF | NF |
| Cast | NF | NF | NF | NF | NF | NF | NF | NF | NF | NF | NF | NF | NF | NF | NF |
| Crystal | NF | NF | NF | NF | NF | NF | NF | NF | NF | NF | NF | NF | NF | NF | NF |
| Miscellaneous | NF | NF | NF | NF | NF | NF | NF | NF | NF | NF | NF | NF | NF | NF | NF |

Table 1. Urinalysis results of diabetic dogs no. 1-3 from day 0-180

N = normal, NF = not found, RBC= Red blood cell, Sq = squamous cells, USG = urine specific gravity,WBC = White blood cell

|  | DM 4 | | | | | DM 5 | | | | | DM 6 | | | | |
| --- | --- | --- | --- | --- | --- | --- | --- | --- | --- | --- | --- | --- | --- | --- | --- |
|  | Day 0 | Day 45 | Day 90 | Day 135 | Day 180 | Day 0 | Day 45 | Day 90 | Day 135 | Day 180 | Day 0 | Day 45 | Day 90 | Day 135 | Day 180 |
| **Dipstick examination** | | | | | | | | | | | | | | | |
| USG | 1.035 | 1.028 | 1.030 | 1.030 | 1.040 | 1.015 | 1.029 | 1.019 | 1.030 | 1.030 | 1.010 | 1.013 | 1.012 | 1.015 | 1.020 |
| pH | 6 | 6 | 6 | 6 | 5.5 | 6 | 6 | 6 | 6 | 6 | 6 | 6 | 6 | 6 | 6 |
| Leukocyte | - | - | - | - | - | - | - | - | - | - | - | - | - | - | - |
| Nitrite | - | - | - | - | - | + | + | + | + | + | - | - | - | - | - |
| Protein | trace | - | - | trace | trace | - | - | - | - | trace | - | - | - | - | - |
| Glucose | 3+ | 3+ | 3+ | 3+ | 3+ | 3+ | 3+ | 3+ | 3+ | 3+ | N | N | N | 1+ | 3+ |
| Ketone | - | - | - | - | - | - | - | - | - | - | - | - | - | - | - |
| Urobilinogen | N | N | N | N | N | N | N | N | N | N | N | N | N | N | N |
| Bilirubin | - | - | - | - | - | - | - | - | - | - | - | - | - | - | - |
| Erythrocyte | - | - | - | trace | - | - | 2+ | - | - | - | - | - | - | - | - |
| **Microscopic Examination** | | | | | | | | | | | | | | | |
| WBC | 2 | NF | 1-2 | NF | NF | 0-1 | NF | 2-3 | NF | NF | NF | NF | NF | NF | NF |
| RBC | NF | NF | NF | few | NF | NF | NF | 1-2 | NF | 0-1 | NF | NF | NF | NF | NF |
| Bacteria | 1+ | few | 1+ | few | 1+ | few | 1+ | 2+ | few | 1+ | few | few | few | few | few |
| Amorphous | NF | NF | NF | few | NF | NF | NF | NF | NF | NF | NF | NF | NF | NF | NF |
| Mucous | NF | NF | NF | NF | NF | NF | NF | NF | NF | NF | NF | NF | NF | NF | NF |
| fungus | NF | NF | NF | NF | NF | NF | NF | NF | NF | NF | NF | NF | NF | NF | NF |
| epithelium | Sq0-1 | Sq0-1 | Sq0-1 | Sq0-1 | NF | Sq0-1 | Sq0-1 | Sq0-1 | Sq0-1 | Sq0-1 | NF | Sq0-1 | Sq0-1 | NF | Sq0-1 |
| Cast | NF | NF | NF | NF | NF | NF | NF | NF | NF | NF | NF | NF | NF | NF | NF |
| Crystal | NF | NF | NF | NF | NF | NF | NF | NF | NF | NF | NF | NF | NF | NF | NF |
| Miscellaneous | NF | NF | NF | NF | NF | NF | NF | NF | NF | NF | NF | NF | NF | NF | NF |

Table 2 Urinalysis results of diabetic dogs no. 4-6 from day 0-180

N = normal, NF = not found, RBC= Red blood cell, Sq = squamous cells, USG = urine specific gravity, WBC = White blood cell
